# The use of a surgical planning tool for evaluating the optimal surgical accessibility to the stapedius muscle via a retrofacial approach during cochlear implant surgery: a feasibility study

**DOI:** 10.1007/s11548-020-02288-8

**Published:** 2020-11-13

**Authors:** Pedro Marquez, Gerd Fabian Volk, Francesca Maule, Daniela Korth, Thomas Bitter, Sven Koscielny, René Aschenbach, Orlando Guntinas-Lichius

**Affiliations:** 1 MED-EL Medical Electronics, Innsbruck, Austria; 2 Department of Otorhinolaryngology, Jena University Hospital, Am Klinikum 1, 07747 Jena, Germany; 3 Department of Radiology, University Hospital Jena, Jena, Germany

**Keywords:** Temporal bone, Dyna-computed tomography, Cochlear implantation, 3D reconstruction, Segmentation, Electrically elicited stapedius reflex

## Abstract

**Purpose:**

During cochlear implant (CI) surgery, visual detection of the stapedius reflex as movements of the stapes tendon, electrically elicited via the CI, is a standard measure to confirm the system's functionality. Direction visualization of the stapedius muscle (SM) movements might be more reliable, but a safe access to the small SM is not defined. A new surgical planning tool for pre-operative evaluation of the accessibility to the stapedius muscle (SM) during a cochlear implantation (CI) via a retrofacial approach was now evaluated.

**Methods:**

A surgical planning tool was developed in MATLAB using an image processing algorithm to evaluate drilling feasibility. A flat-panel computed tomography (CT) combining a rotational angiographic C-arm units with flat-panel detectors (Dyna-CT) was used. In total, 30 3D Dyna-CT-based temporal bone reconstructions were evaluated by automatized algorithms, generating a series of trajectories and comparing their feasibility and safety to reach the SM via a retrofacial approach. The predictability of the surgical planning tool results was tested in 5 patients.

**Results:**

The surgical planning tool showed that a retrofacial access to the SM would be feasible in 25/30 cases. Moreover, the evaluation of the predictability of the results obtained with the surgical planning tool conducted during 5 CI surgeries confirmed the results. Both the surgical planning tool and the results on SM accessibility via retrofacial approach during CI showed that this is safe and feasible only when the SM-exposed area was > 25% of its total, the distance between the SM and the facial nerve was > 0.8 mm, and the surgical corridor diameter was > 3 mm.

**Conclusion:**

The surgical planning tool seems to be useful for the pre-operative evaluation of the accessibility to the SM during a CI surgery via a retrofacial approach. Further prospective studies are needed to validate the results in larger cohorts.

**Electronic supplementary material:**

The online version of this article (10.1007/s11548-020-02288-8) contains supplementary material, which is available to authorized users.

## Introduction

The temporal bone is a complex structure housing small and functionally very important structures like the middle-ear including the ossicles; the inner-ear including the cochlea, vestibule and semicircular canals; the bony canals for the facial and vestibulocochlear nerves; and the related vasculature. The middle-ear houses the stapedius muscle (SM), the smallest muscle of the human body, the belly of which has a length of only 2–4 mm [1]. The SM contracts in response to loud sound stimuli, and this involuntary movement is known as stapedius reflex. Its function is to protect the inner-ear against damages caused by exposure to excessive noise. Normal hearing people have a stapedius reflex threshold (SRT) at about 70–100 dB sound pressure level. During cochlear implant (CI) surgeries, visual detection of the stapedius reflex electrically elicited via the cochlear implant (electrically elicited stapedius reflex; eSRT) is considered a standard outcome to confirm the system's functionality [2]. However, the reduced size of the SM makes the detection of such a movement very difficult if not sometimes impossible. An effective alternative to visual detection is given by electromyographic (EMG) recording of the SM contraction [2]. While this technique is very reliable, it requires the EMG electrodes to be effectively and safely placed on the SM. Recently, we showed that retrofacial access to the unbent distal part of the SM, planned in advance and based on the analysis of high-resolution computed tomography (CT) datasets of the temporal bone and 3D reconstructions complies with these requirements [3]. To realize a resolution to visualize the configuration of the SM in detail, Dyna-CT datasets were used. Beyond its high spatial resolution of temporal bone structures, Dyna-CT allowed a fast acquisition of 3D datasets with relative low radiation dose [3]. The retrofacial approach corresponds to drilling posteriorly to the FN, rather than anteriorly as in the facial recess approach normally used on CI surgery. Pre-operative planning tools are of paramount importance for any minimal-invasive otosurgical approach, particularly for minimal-invasive robotic middle-ear surgeries, in order to prevent the damage of functional structures of the ear [4–6], supporting the conclusion of previous works describing the use of newly developed software for automated segmentation and trajectory planning for temporal bone surgery [7]. The present study describes the implementation and use of a new surgical planning tool designed to evaluate and plan the access to the SM via drilling of the temporal bone in otosurgery in a safe and effective way.

## Methods

### Study design and patient data

In total, 30 Dyna-CT images of the temporal bone and 5 cochlear implant surgery protocols were collected within a surgical planning tool prospective observational study including 30 patients (15 women and 15 men; 61 ± 20 years). The datasets are the same previously analyzed in [3]. The institutional ethics committee approved the study protocol (no. 4896-08/16) in 2016, and the data collection spanned between June 2016 and May 2018. The images were generated by the Department of Radiology within the routine clinical examination of these patients. Dyna-CT was performed to verify the following indications: (a) cochlear implantation (23 cases), (b) middle-ear implantation (1 case), or to confirm 5 cases of chronic otitis media and 1 case of exostosis of the external auditory canal. Dyna-CT images were acquired in 17 cases only on the right, in 10 cases only on the left and in 3 cases on both sides of the head. The images were completely anonymized before undergoing the processing through our surgical planning tool.

### Inputs: Dyna-CT imaging, segmentation and 3D reconstruction

The tool described in this work requires as input a set of 3D surface files in STL format, product of segmentation and 3D reconstruction of anatomical imaging data. For the present study and due to the numerous advantages of Dyna-CT in otology, the acquisition was performed as recently described in [3]. Briefly, Dyna-CT is a special flat-panel computed tomography combining a rotational angiographic C-arm units with flat-panel detectors. Volumetric data were acquired with a single rotation of the C-arm mounted flat-panel detector cone-beam CT system (Artis Zeego Q system, Siemens Medical Solutions, Forchheim, Germany) and reconstructed using Dyna-CT. Post-processing of the volumetric data was achieved using Syngo 4D software (Leonardo, Siemens, Forchheim, Germany). Post-processing included an automatic reconstruction of the volumetric dataset for each patient as an axial plane dataset consisting of 400–550 slices (512 × 512 imaging matrix), a slice thickness of 0.2 mm, slice separation of 0.5 mm resulting in a voxel size of 0.2 × 0.2 × 0.2. From this isotropic axial dataset paracoronal and parasagittal planes were reconstructed parallel and perpendicular to the petrous bone axis to evaluate middle- and inner-ear structures routinely.

Segmentation of different anatomical structures was performed with the 3D Slicer software (free download at www.slicer.org) using a standardized mostly manual method. Afterward, 3D reconstructions were exported as single STL files using the RAS coordinate system and limited to 10,000 faces. The segmentation method corresponds to threshold-masked painting primarily on axial slices, with corrections and details on coronal and/or sagittal slices [3]. Additionally, semi-automatic methods such as pure thresholding was used for the temporal bone, and morphological contour interpolation (‘fill between slices’ tool in Slicer 3D) was used for the sigmoid sinus. Prior to segmentation, an initial histogram normalization was performed [3]. This means a reference dataset was selected whose maximal and minimal gray values were closest to the average among all datasets, and subsequently all other datasets were normalized to the range of gray values of the reference. Threshold values for each structure were selected empirically and are specified in Supplement Table 1. The time needed by an experienced person to segment one middle-ear dataset was of about 2 h [3].

The structures evaluated by means of the surgical planning tool were: (a) the stapedius muscle (SM), (b) the vestibular-cochlear system (VC), (c) the facial nerve (FN; as a minimum including the mastoid segment with or without the chorda tympani), (d) the sigmoid sinus (SS) and (e) the surface of the temporal bone in the mastoid region (TB). Other structures, such the round window (RW), the ossicles (OS), the external ear canal or other ear structures, were optionally included only for improved visualization purposes in some datasets, but they were not considered by the tool during the calculation of trajectories

### Surgical planning tool: evaluation of orientations and optimal trajectory

**Fig. 1:**
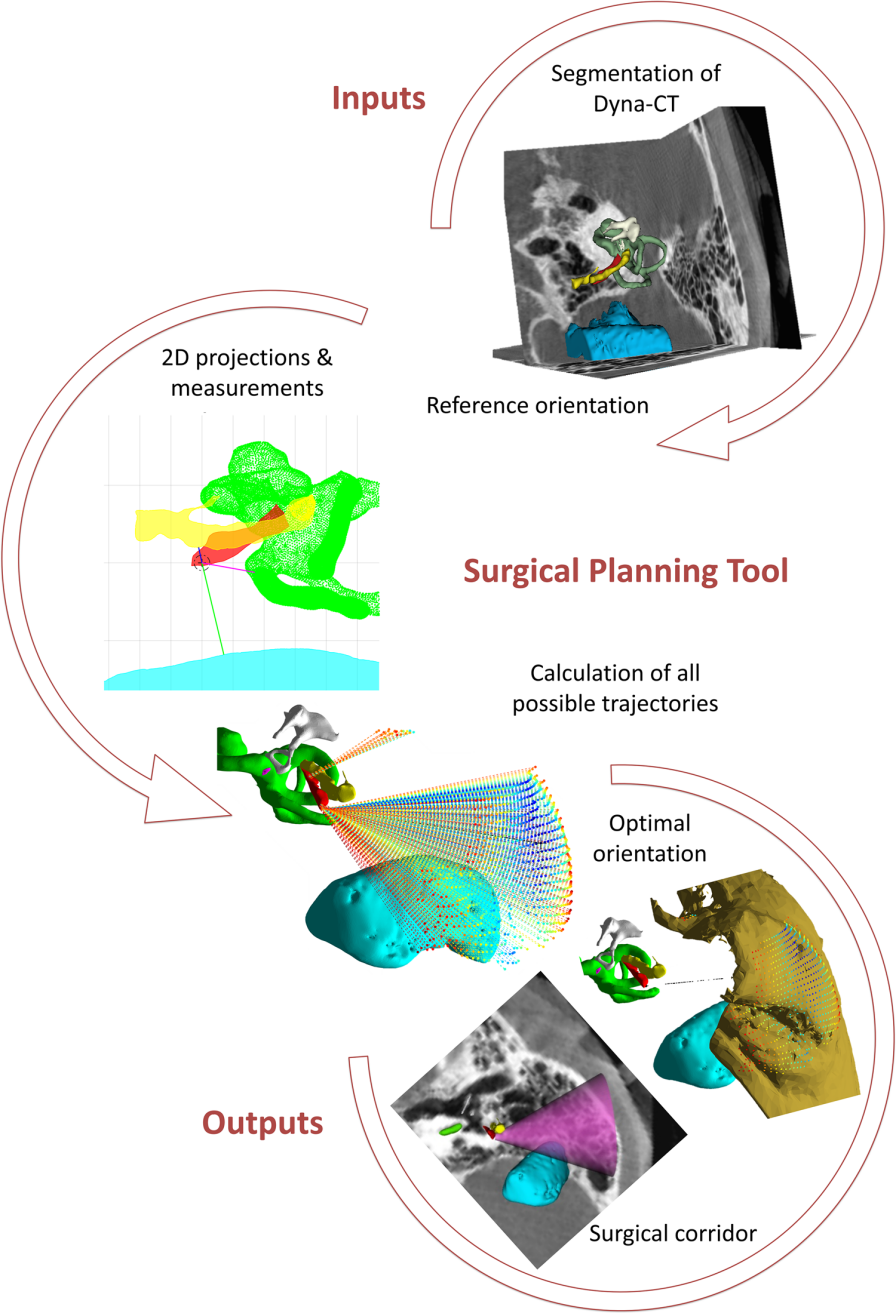
Overview of the procedure used for the automatic evaluation of retrofacial access to the SM by means of the surgical planning tool. 3D reconstructions of the anatomical structures made from Dyna-CTs are processed to generate 2D projections and analyzed by the surgical planning tool in order to select the best orientation and automatically generate the best surgical corridor to access the SM via retrofacial approach

A schematic description of the main steps required to automatically generate the optimal surgical corridor, to access the stapedius muscle via retrofacial approach, by means of the surgical planning tool is depicted in Fig. 1. This image processing-based tool was developed on MATLAB (version 2018b, The MathWorks Inc., Natick, USA). It requires as input the 3D surface reconstructions of the relevant anatomical structures as previously described and a few numerical parameters such as the range of rotations, step, drill bit size and minimum safety distance. Details are presented further in this section. The tool provides as outputs a 3D surface representing the safe surgical corridor and a set of measurements that may be used as guidelines during the intraoperative procedure.

1. Reference orientation

**Fig. 2:**
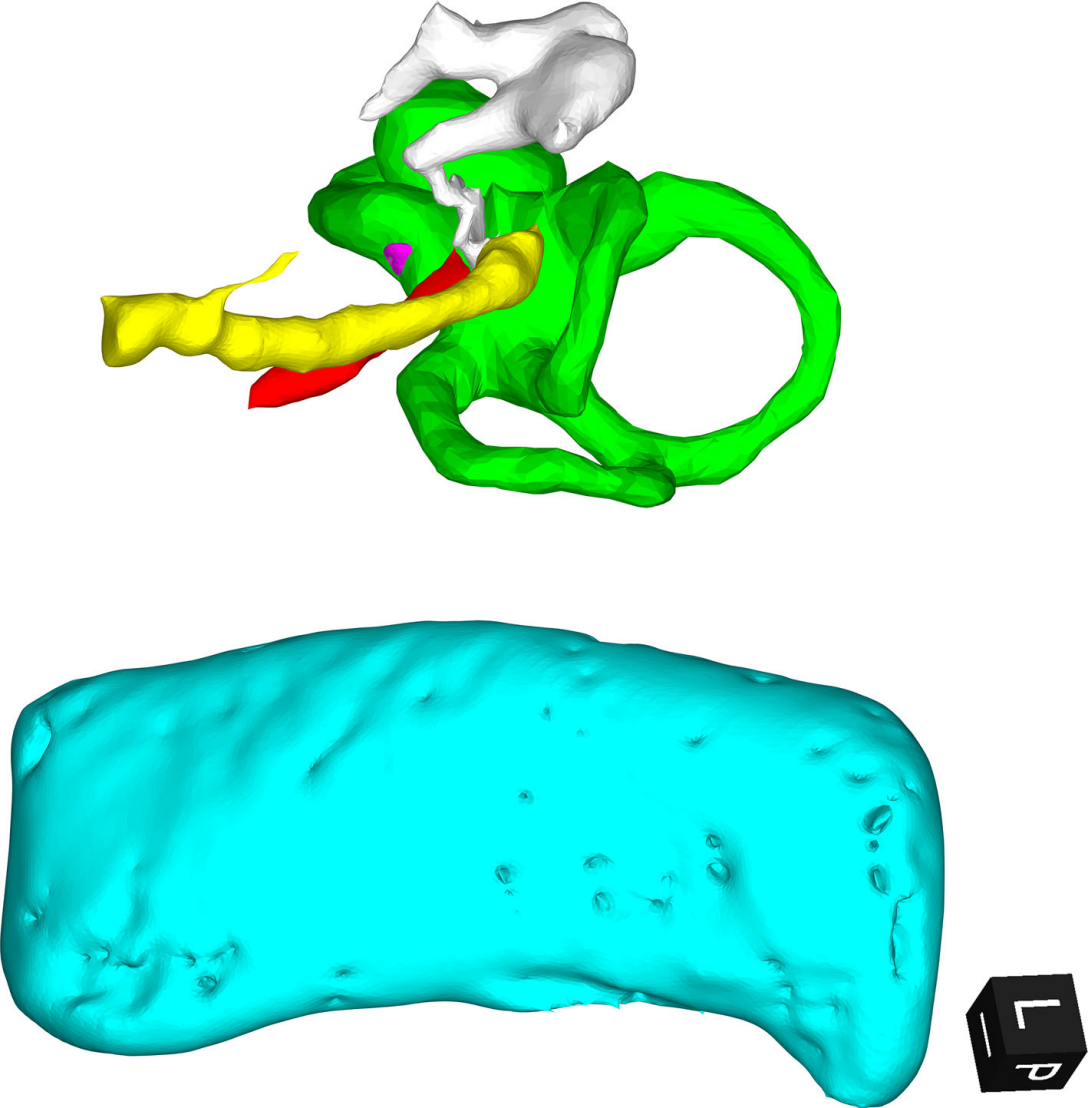
Orientation of the 3D reconstructions from the point of view of a surgeon performing a posterior tympanotomy during a routine cochlear implantation. Red = stapedius muscle (SM); yellow = facial nerve (FN); light blue = sigmoid sinus (SS); green = cochlea and vestibular system (VC): magenta = round window; white = ossicles. The cube at the bottom right gives the orientation: L = lateral; P = posterior; I = inferior

The first analysis step is the only one that requires an input from the user. Essentially, a 3D picture of the input surfaces is shown in a lateral to medial view, automatically calculated based on the original DICOM files orientation and the specified surgery side. Then the user may manually rotate this picture using 2 different axes. The goal here is to orient the picture toward the point of view of a surgeon performing a posterior tympanotomy during a routine cochlear implantation. This corresponds to a patient’s head in a supine position and slightly laterally rotated, with a view from lateral to medial on the surgery side. This orientation is better depicted in Fig. 2. Once the desired orientation is set, it is taken as reference for the subsequent process. The selection of this reference orientation is merely to facilitate the visualization and initialize the process with a reference system relatable and understandable to the surgeon. In fact, the final outputs and tool performance are not influenced by the initial orientation, since the algorithm that calculates the access route does not take this orientation as a parameter, but rather as the origin of a coordinate system to which results can be referenced.

2. Calculation of all possible trajectories

The next step corresponds to the automatic evaluation of potential access routes to the SM using ideal linear trajectories. These shall start from the mastoid bone outer surface and end on the most proximal surface of the SM. The automatic evaluation is performed by stepwise rotating the 3D image around two axes and assessing for each rotation a potential linear trajectory. Rotations are done within a range and step values previously specified as parameters and taking as reference the initially set (posterior tympanotomy) orientation. The orientation range values used for processing the data of this study were − 30° to + 30° around the ‘roll’ or anterior–posterior axis (cervical lateral flexion) and − 40° to + 20° of head tilt around the ‘yaw’ or inferior–posterior axis (cervical rotation), with steps of 2° in between. This range of values is within a normal and practical range of motion of the neck [8]. Steps of 2° were chosen empirically to keep a balance between a significant number of orientations to be analyzed and a short time for running all necessary computations.

3. 2D projections and measurements

**Fig. 3:**
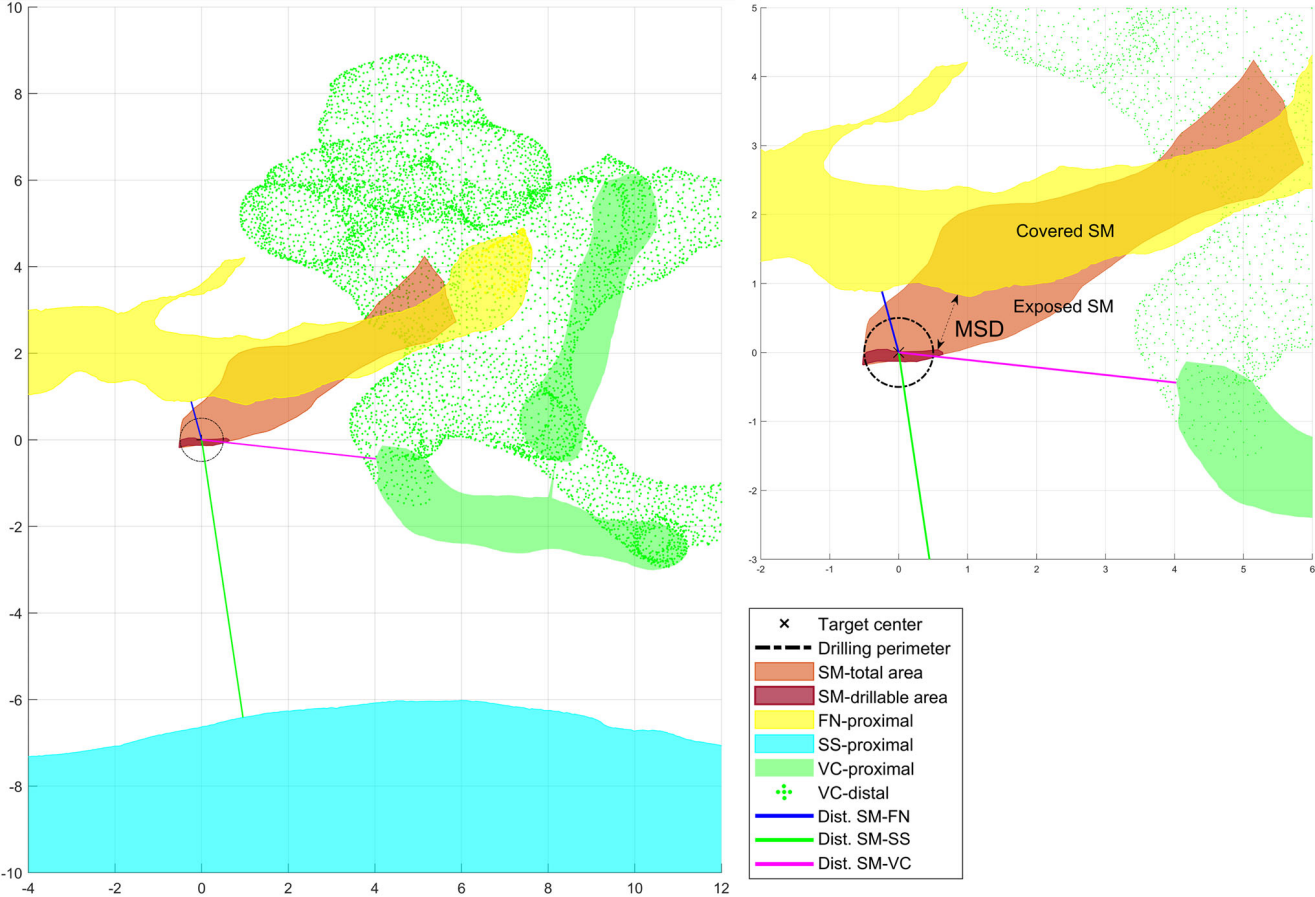
2D projection and measurements on the middle-ear anatomy for one particular orientation. The upper right picture shows a close-up look on the SM total and ‘drillable’ area (keeping the MSD margin). The target center (cross), drilling perimeter (dot-dash line) and minimum safety distance MSD (arrow) are also displayed. Information about the position of the proximal and distal parts (polygons) of the following structures is also provided: yellow = facial nerve (FN); light blue = sigmoid sinus (SS); green = cochlea and vestibular system (VC). The axes of the image are centered on the target center on the SM (red) and measured in mm

A 2D projection of the rotated 3D image is generated at each step within the range of orientations. This corresponds to the projection of the 3D surfaces on a plane formed by the two axes of rotation. In other words, it emulates in 2D the ‘surgical view’ that a surgeon would have through the microscope at that specific orientation (Fig. 3). The projection is computed by simply rotating the 3D surfaces according to the two axes and zeroing one of its three coordinates, which is related to the depth from the current point of view. This depth value, however, is evaluated for each point in each structure to verify whether it lies proximal or distal with respect to the stapedius muscle. From each projection, a set of seven polygons are computed: (1) projected SM, (2–4) proximal sections of FN, SS and VC and (5–7) distal sections of the FN, SS and VC. This helps evaluate as shown in Fig. 3, which structures lie in front of the SM, thus potentially blocking the access.

An important aspect needs to be taken into consideration for all these computations, which is the error introduced by the Dyna-CT imaging technique and segmentation. Therefore, a minimum space between any potential drilling target and the borders of other anatomical structures (FN, VC, SS) must be kept. Accordingly, a ‘minimum safety distance’ (MSD) parameter is introduced and defined as half of the drill tip size (drill radius) plus the above-mentioned error (${\text{MSD}} = r_{\text{drill}} + \varepsilon$). For the purposes of this study, an error value of 0.2 mm was selected, based on the slice thickness of Dyna-CT, and similar error considerations used for image-guided CI surgery as in [4]. It should be noted that this value is also dependent on the manual segmentation error and the intraoperative system used for drilling and may be changed accordingly. Common drill sizes used in otosurgery range between 0.6 and 7 mm. The drill size selected for this study corresponds to a standard size of 1.2 mm, commonly used for posterior tympanotomy [9]. These considerations lead to a MSD of 0.8 mm (0.6 mm drill tip radius + 0.2 mm error).

Subsequently, each 2D projection and set of polygons are used to determine the feasibility of the respective access-route orientation, based on the assessment of the following measurements:

- Size of the SM: this corresponds to the geometrical 2D area of the projected SM polygon (1).
- Exposed SM: corresponding to the portion of SM that is not covered by proximal segments of other structures. It is another polygon computed by subtracting from the SM polygon (1), all others (2–7).
- Exposure of the SM: calculated as the ratio between the geometrical 2D area of the exposed SM (b) and the size of the SM (a).
- Target center: corresponds to the ideal target to be reached by the center of the drill bit. First, the ‘drillable’ portion of the SM is computed by subtracting from the exposed SM polygon (b), a margin with other structures based on the ‘minimum safety distance’. Then, the target center is calculated as the centroid of this SM drillable area.
- Distances between the ‘target center’ and other structures (FN, SS and VC). Calculated as the minimum Euclidean distances between the target center coordinates and all point inside each of the other polygons.

All projections polygons and measurements are better depicted in Fig. 3.

4. Optimal orientation

The following step corresponds to determining the ‘optimal trajectory’ among all previously computed rotations and considering the measurements on each projection. This is as an optimization problem where a cost function is minimized for a set of parameters among all possible trajectories. Such an optimization problem could be tackled by a machine learning approach [10, 11], but this would require enough training data which unfortunately is not available for this application. A much simpler approach is used by the surgical tool by defining a ‘safety measure’ $S\left( i \right)$ for each trajectory $i$:

$$ S\left( i \right) = \alpha *\frac{{{\text{ASM}}\left( i \right)}}{{\hbox{max} \;{\text{ASM}}}} + \beta *\frac{{{\text{DFN}}\left( i \right)}}{{\hbox{max} \;{\text{DFN}}}} + \gamma *\frac{{{\text{DSS}}\left( i \right)}}{{\hbox{max} \;{\text{DSS}}}} + \delta *\frac{{{\text{DVC}}\left( i \right)}}{{\hbox{max} \;{\text{DVC}}}} - \theta *\frac{{{\text{Roll}}\left( i \right)}}{{\hbox{max} \;{\text{Roll}}}} $$

where

- ${\text{ASM}}$ is the ratio between the exposed and the total area of SM (b);
- ${\text{DFN, DSS, DVC}}$ are the distances between the exposed area of the SM and the proximal portion of the FN, SS and VC, respectively (e); and
- ${\text{Roll}}$ is the angle of rotation around the anterior–posterior axis.

**Fig. 4:**
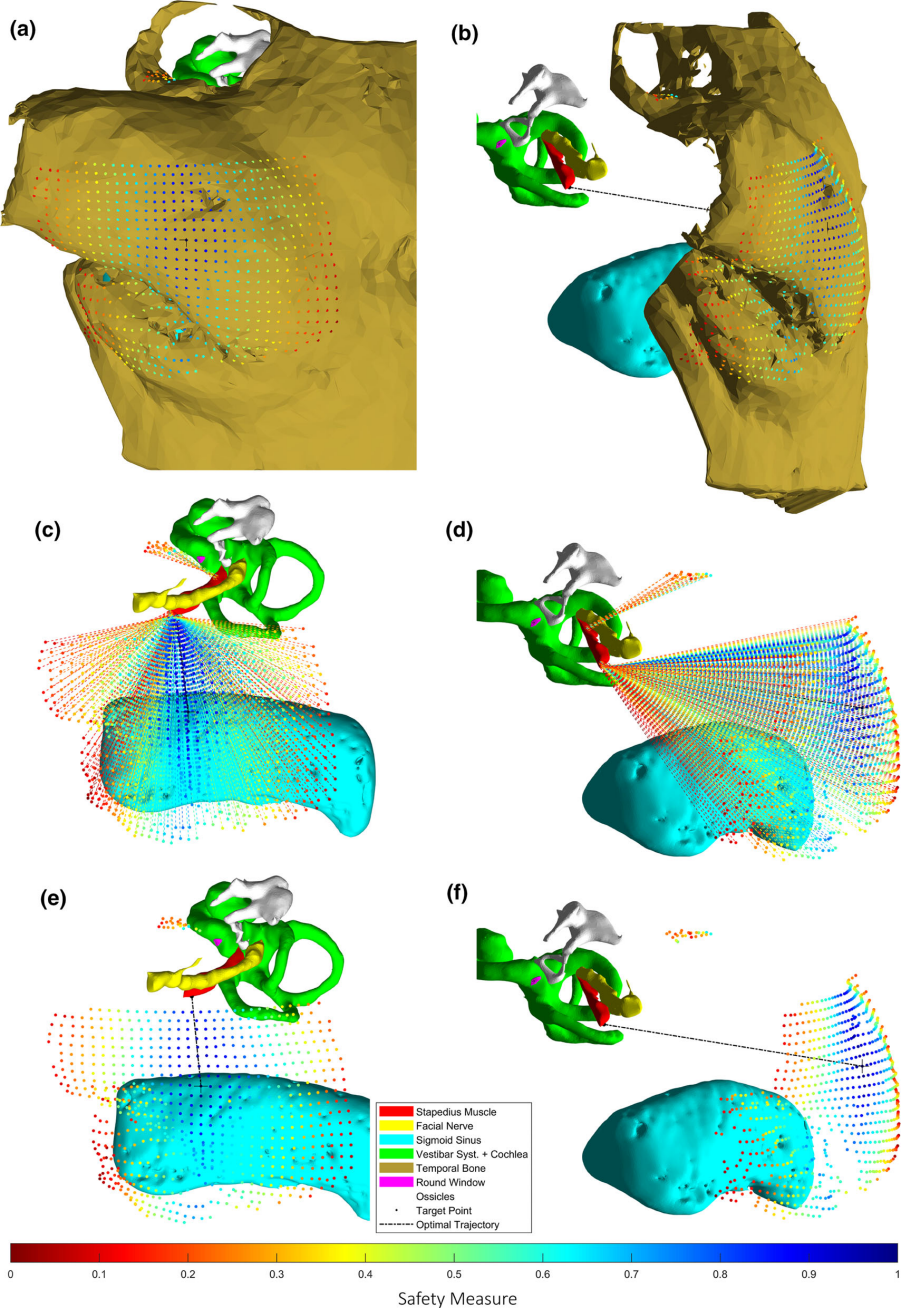
Automatic calculation of all the possible trajectories starting from the skull. **a** and **b**: skull map: 3D visualization of all the possible trajectories projected onto points on the skull; **c** and **d** visual display of all the generated trajectories, by means of a color scale where blue represents the safest and most effective and red the least safe and most complex access route to the SM; **e** and **f**: display of the optimal access route and the starting point of all the other trajectories

The coefficients $\alpha , \beta ,\gamma ,\delta , \theta$ represent the weight of each of these properties toward the safety of each trajectory. Their values (0.6, 0.6, 0.6, 0.3 and 1.2, respectively) were determined empirically, due to lack of enough data and ‘correct outputs’ for a mathematical approach. However, they were defined following knowledge shared by otosurgeons on the subject; for example giving higher weight to the exposed SM area and distances, and setting a negative weight on how much rotation is needed from the initial orientation. This function may be considered as the simplest approach to automatically analyze all possible trajectories and define their feasibility and safety based on a quantitative rather than merely qualitative scale. For visualization, the surgical planning tool displays all possible trajectories in a 3D representation. Each starting point is projected on the skull and color coded according to the safety profile. Moreover, the calculated optimal trajectory is shown with a black dotted line (Fig. 4).

Based on their experience, the surgeons may use the automatically calculated optimal access route or evaluate an alternative. This can be done by selecting another of the generated trajectory starting points in the 3D image. Furthermore, other measurements such as the depth of the SM distal to the FN along the drilling depth, the distance between the mastoid bone and the target center, and the degrees of rotation in the yaw axis, are also computed and shown for analysis. Even though these extra measurements may provide additional information for a manual selection of the optimal access route, they were left out from the equation for the purposes of this study, as they were considered less relevant for an automatic optimization method.

### Outputs

#### Surgical corridor

All the automatic calculations by the surgical planning tool described above assume a perfectly linear trajectory between the skull and the target center in the stapedius muscle. In real temporal bone surgery, the decision of the surgeon on the best surgical drilling path, in terms of safety and effectivity, is based on their experience and on the visual evaluation of the relevant anatomical structures during surgery. Additionally, surgeons might also consider other patient-specific characteristics that may get lost in a pure image processing-based analysis. In particular, the drilling path that a surgeon manually follows may be better described by a cone-like space rather than by a straight-line trajectory, as it would be for example the case of robotic drilling.

**Fig. 5:**
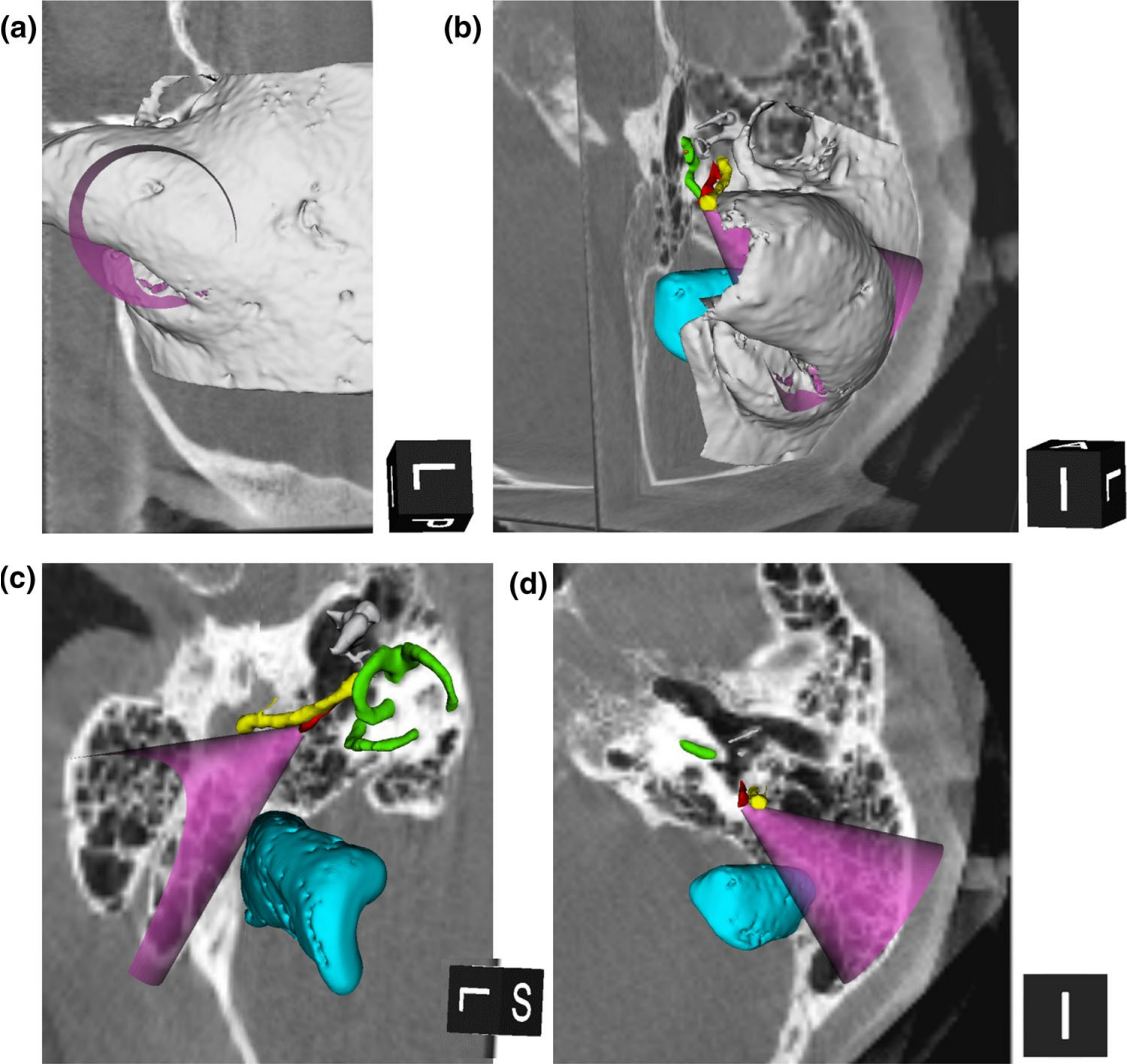
The automatically generated cone-shaped surgical corridor (purple) is superposed onto the 3D reconstruction and Dyna-CT axial and sagittal slices. **a** and **b** show the temporal bone surface, whereas **c** and **d** show the corresponding inner structures, with the cone-shaped surgical corridor (purple) positioned with the tip on the target center. The cubes at the bottom right of each picture give the orientation: L = lateral; P = posterior; I = inferior

Upon selection of the optimal trajectory, the surgical planning tool generates the drilling ‘surgical corridor’ in the shape of a truncated cone. The small base of this cone is positioned onto the target center and has a diameter defined by the drill tip size. The axis of the cone is centered on the optimal linear trajectory between TB and SM. The angle of the cone is maximized so that the curved surface of the cone reaches the minimum safety distance from the FN, SS or VC. Visualizing the access route in a cone shape provides a more intuitive representation than an imaginary straight line, showing a more realistic scenario for manual drilling of the temporal bone. The cone (surgical corridor) can be exported as an STL file and overlaid onto the 3D reconstructions and/or CT images (in slicer 3D) for assessment by the surgeon. Figure 5 shows that a very broad drilling cone would result in case of a well-exposed SM.

#### Surgical guidelines and measurements

**Fig. 6:**
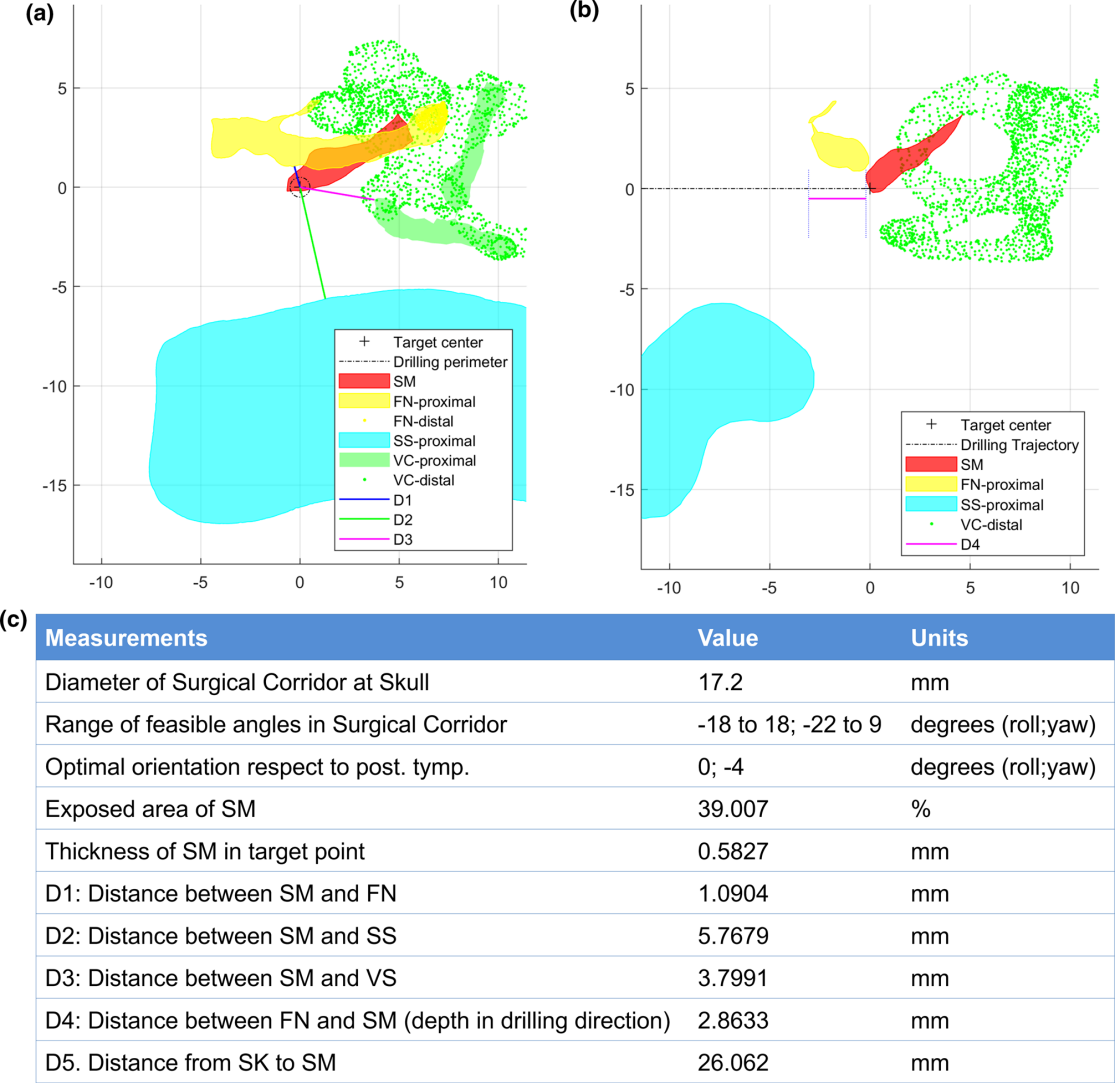
Display of the output information from the surgical planning tool showing **a** the 2D projection from the point of view of the surgeon drilling via the optimal trajectory toward the SM; **b** a 90°-rotated view (inferior–superior) of the same picture with depth information; and **c** a table generated with summary of measurements and surgical guidelines for this example case study

In addition to the optimal trajectory and the surgical corridor, the surgical planning tool provides a summary of all the properties calculated during the process as these may be helpful for pre-operative analysis as well. This includes: the diameter of the surgical corridor at the surface of the skull, range of feasible orientation angles, 2D projections based on the optimal trajectory, exposed area of SM, thickness of the targeted portion of the SM, distance between the SM and the FN, SS, VC, respectively, depth of the SM distal to the FN on the drilling direction and distance between the TB and the SM on the drilling axis (see Fig. 6).

### Application of the surgical planning tool

The surgical planning tool was employed to evaluate the possibility to access the SM with a retrofacial approach using 30 Dyna-CT image datasets of the temporal bone and additionally during and after 5 cochlear implantations [3]. In this case, the surgeon performing the cochlear implantation was not part of the evaluation team of the Dyna-CT images of the respective patients, and the evaluating team did not know about the results of the retrofacial approach to access the SM during the CI at the time of the evaluation of the Dyna-CT images.

## Results

### Application of the surgical planning tool to the study of Dyna-CT images

The surgical planning tool was used to evaluate 3D reconstructions of 30 Dyna-CT images. The complete automatic processing of one dataset using the tool takes 14.98 ± 7.40 min, on an Intel Core i7 processor machine @3.4Ghz with 16 GB RAM. The results of the analysis are provided in Table 1. In short, considering a retrofacial approach, the SM could have been exposed in 25/30 cases (83%). For the feasible cases, the mean distance between the SM and the mastoid segment of the facial nerve was 0.99 ± 0.37 mm, while the mean distances between the SM and the sigmoid sinus (4.58 ± 2.60 mm) or the vestibular-cochlear system (3.55 ± 1.72 mm) were longer. Using a retrofacial access, the SM would have been placed 1.69 ± 1.19 mm behind the facial nerve, and the otosurgeon would have had a surgical corridor of about 10.58 ± 7.44 mm of diameter at the surface of the skull.

**Table 1:** Use of the surgical planning tool to generate an optimal retrofacial access route to the SM in 30 3D Dyna-CT scans

Parameter	Measurable in *N* patients	Mean	SD	Min	Max
Percentage of exposed SM area, for the orientation defined optimal by the surgical planning tool	30	50.03	31.00	0.00	100.00
Mean percentage of exposed SM area, across all possible orientations generated by the surgical planning tool	25	55.84	22.05	20.00	100.00
SD of percentage of exposed SM area	25	12.32	5.80	0.00	22.00
Distance SM-FN in mm	25	0.99	0.37	0.09	2.03
Distance SM-SS in mm	25	4.58	2.60	1.00	10.13
Distance SM-VC in mm	25	3.55	1.72	1.35	7.77
Depth of SM behind FN in mm	25	1.69	1.19	0.08	4.71
Optimal rotation (roll) in degrees	25	− 0.16	8.24	− 22.00	28.00
Optimal head tilt (yaw) in degrees	25	− 7.84	16.46	− 40.00	20.00
Diameter of surgical corridor at the level of the mastoid bone, in mm	25	10.58	7.44	2.00	27.20

*SD* standard deviation, *SM* stapedius muscle, *VC* vestibular-cochlear system, *FN* facial nerve, *SS* sigmoid sinus (SS)

### Confirmation of the surgical planning tool results during CI surgery

**Fig. 7:**
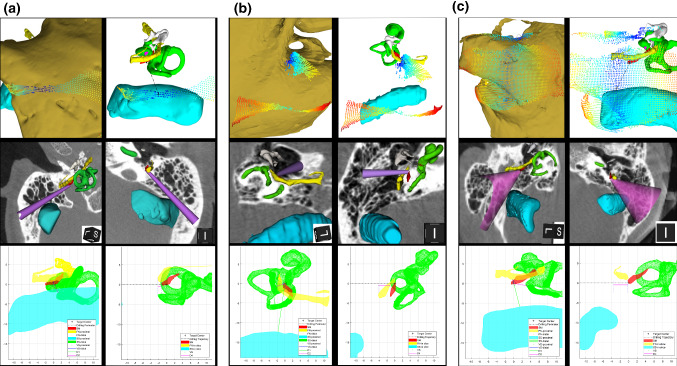
Predictability of the SM accessibility via retrofacial approach based on the surgical planning tool results tested during 5 cochlear implant surgeries (details on the cases in: [3]). **a** Patient 2: the predicted limited feasibility of access was confirmed during surgery; **b** Patient 3: the predicted unfeasibility of access was confirmed during surgery; **c** Patient 5: the predicted high feasibility and easiness of access were confirmed during surgery

The predictability of the results obtained in the assessment of Dyna-CT images by means of the surgical planning tool was tested during 5 cochlear implant surgeries. In these cases, the accessibility of the SM via a retrofacial approach according to the optimal trajectory generated by the surgical planning tool was compared with the true accessibility to the SM via a retrofacial approach during CI. The results are shown in Table 2 and in Fig. 7. In short, when the SM exposed area was > 40% according to the surgical planning tool (Patient 1 and 5), the access to the SM via retrofacial approach was easily achieved during the surgery. As expected, when the SM exposed area was > 25 but < 40% according to the surgical planning tool, the access to the SM via retrofacial approach was achieved with difficulties during the surgery (Patient 2 and 4). In all the four cases, the distance between the SM and the FN was > 0.8 mm and the surgical corridor diameter was > 3 mm. In the case of Patient 3 the access to the SM via retrofacial approach was considered unfeasible by the surgical planning tool and could not be achieved during the surgery. In this specific case, even if the exposed SM area was about 30% (on the facial recess, not posterior to FN), the distance between the SM and the FN was 0.55 mm and the surgical corridor had a diameter of 2 mm.

**Table 2:** Predictability of the SM accessibility via retrofacial approach based on the surgical planning tool results tested during 5 cochlear implant surgeries

Patient	#1	#2	#3	#4	#5
Predicted feasibility of the access to the SM via a retrofacial approach	Y	Y	N	Y	Y
Percentage of exposed SM area	40.79	37.08	29.84	25.23	49.79
Thickness of SM at target point in mm	0.44	0.98	0.48	0.23	0.99
Distance SM-FN in mm	1.00	0.82	0.55	0.89	1.05
Distance SM-SS in mm	3.94	1.48	12.64	5.07	3.32
Distance SM-VC in mm	3.78	3.66	2.37	5.42	3.52
Depth of SM behind FN in mm	0.74	1.26	3.02	1.08	1.97
Range of feasible angles of rotation (roll) at surgical corridor in degrees	− 14 to 18	− 4 to 4	6	− 16 to 8	− 16 to 16
Range of feasible angles of head tilt (yaw) at surgical corridor in degrees	− 16 to 16	− 28 to − 26	16	− 20 to 0	− 36 to − 4
Optimal orientation with respect to posterior tympanotomy in degrees (roll; yaw)	2; 0	0; − 26	6; 16	− 2; − 8	0; − 20
Diameter of the surgical corridor at the plane of the mastoid in mm	23.2	4.40	2.00	19.20	16

The parameters refer exclusively to the trajectory that the surgical planning tool selected as ‘optimal’

*SM* stapedius muscle, *VC* vestibular-cochlear system, *FN* facial nerve, *SS* sigmoid sinus (SS)

## Discussion

The development of accurate surgical planning tools including algorithms to define optimal trajectories to a target small structure in the temporal bone becomes more and more important since (1) they would strongly reduce the invasiveness of the surgical approach minimizing the risks of damage of small but functionally important structures in the temporal bone, and (2) they are the natural prerequisites for automated robotic strategies, such as robotic CI surgery [4, 5, 7]. Our study presents the preliminary findings on the use of a new surgical planning tool, using as primary input 3D reconstructions of specific middle-ear structures, from pre-operative Dyna-CT scans.

The main use of the surgical planning tool described in this work, is the generation of an effective and safe access route to the SM via a retrofacial approach, with main focus on traditional surgery, which is not necessarily the case on similar trajectory evaluation algorithms that aim instead toward robotic or automated surgery [5, 7]. In fact, the use of this surgical planning tool may improve traditional surgery by providing the surgeon with an enhanced visualization of the middle-ear structures from the actual surgical point of view, thus allowing an optimal planning of the access. In addition, the current surgical planning tool may be translated into a robotic surgery scenario and/or combined with more complex techniques, such as deep-learning algorithms, that have already been successfully implemented in other trajectory calculation methodologies [12]. Minimal invasiveness is an important topic for otosurgery. Robotic surgery is the future for many otosurgical procedures. Hence, modern surgical planning tools for surgical safety and more predictive results become more and more important [4–6].

Presently, the surgical planning tool would particularly profit from the implementation of algorithms supporting at least a semi-automatic segmentation of the middle- and inner-ear structures [4, 7]. Automatic segmentation could reduce the processing time and facilitate the use of this tool in clinical practice and would potentially reduce the variability of the results obtained manually, due to the significant interindividual variability and the reduced size of the said structures. Another limitation of the current tool is the need for a manual selection of the initial orientation, since there is no implemented check on whether the selected orientation actually corresponds to a posterior tympanotomy view. As a proposed improvement of the tool, a check on this selection can be implemented by for example ensuring that either most of the points in the FN, or alternatively its centroid, are positioned proximal to the points or centroid of the SM. Likewise, an automatic selection of the initial orientation can also be implemented. Nonetheless, for the purpose of this study a manual selection was preferred in order to enable the surgeon to freely visualize and orient themselves in the scene, as they would do with the microscope in the surgical room.

In this work, we could show that the surgical planning tool results have a high predictability even in a small sample size. The evaluation of 30 3D Dyna-CT images by means of the surgical planning tool showed that the accessibility to the SM via a retrofacial approach is expected to be feasible in more than 80% of the cases. The predictability of this results was as well verified and confirmed in 5 patients during cochlear implantation. Moreover, all results obtained by the surgical planning tool in terms of surgical access predictability were congruent to those achieved via a manual and qualitative evaluation in [3] using the same datasets.

A clinically relevant aspect of the surgical planning tool is its ability to give a clear idea about the tininess of structures such as the SM. Using microCT data, Wojciechowski measured an average length of 2.98 ± 0.51 mm and width of only 1.26 ± 0.29 mm for the belly of the SM. However, they could not differentiate between its bent and unbent portion. Thus when the latter is the target of a surgical approach, as it was our case [3], this type of measurements is not sufficiently accurate and safe. Indeed, the evaluation of Dyna-CT images by the surgical planning tool showed that exactly determining the exposure of the unbent portion of the SM belly is an essential prerequisite for a safe and accurate access to said muscle via retrofacial approach. Furthermore, the bent form of the SM and its relation to the facial nerve is the primary reason for a better exposition via the retrofacial approach. An access to this part of the SM is not feasible in most cases via the facial recess. Our initial results seem to confirm that this parameter is far more important than the width of the corridor and the distance between the FN and the volume of the SM behind it (Supplement Table 2).

With the surgical planning tool, we could show that on average the distance between the SM and the mastoid segment of the FN is less than 1 mm. The FN is especially at risk during exposure of the SM via a retrofacial approach, because the belly of the SM is located on average 1.69 ± 11.9 mm deeper than the FN, i.e., it is mostly behind it from the point of view of the surgeon. Thus, the implementation of the results obtained with the surgical planning tool during the surgery planning would improve the effectiveness of the approach as well as its safety. The preliminary results of the presented pilot study confirmed the predictability of the surgical outcomes. Due to the small sample size, the results are to be interpreted with caution and need confirmation in a subsequent study with larger sample size. Also, the surgical planning tool can evaluate a facial recess approach in case it is of interest. This and its combination with intraoperative image-guided navigation would further promote the use of the surgical planning tool for both traditional surgery and robotic approaches [5, 6, 13].

A limitation of the present study is that only 3D Dyna-CT images were used as input for the surgical planning tool. While this type of images is considered the current state-of-the-art technique to capture the tiny structures of the middle-ear, it is not widely used during the pre-operative preparation for CI surgeries, since standard CT is often preferred to this type of image acquisition. Future prospective studies should be considered to evaluate whether standard CT could represent a sufficiently good input for the surgical planning tool.

For the purposes of this work, we exclusively performed the segmentations and the 3D reconstructions with the 3D Slicer software. However, the use of any other DICOM viewer and segmentation software would be compatible with the use of the surgical planning tool. Currently, the full set of calculated parameters is presented to the surgeon. One next step will be to present the tool to a larger group of otosurgeon and to analyze the serviceability in detail.

## Conclusions

We developed and used a new surgical planning tool for the pre-operative evaluation of the accessibility to the SM during a CI surgery via a retrofacial approach. The results obtained with the surgical planning tool in a small case series showed in theory a high predictivity in the otosurgery field. Additional improvements of the tool may be implemented with help of more data availability and inclusion of automatized segmentation and optimization methods. Similarly, further prospective studies shall be considered to validate the results of this work in larger cohorts in order to implement the use of the surgical planning tool in clinical routine.

## Electronic supplementary material

Below is the link to the electronic supplementary material.

Supplementary material 1 (DOCX 49 kb)

## Data Availability

The funders had no role in study design, data collection and analysis, decision to publish or preparation of the manuscript.
