# Supplementary material for: The use of a surgical planning tool for evaluating the optimal surgical accessibility to the stapedius muscle via a retrofacial approach during cochlear implant surgery: a feasibility study
Source: Int J Comput Assist Radiol Surg. 2020 Nov 13;16(2):331–43. doi: 10.1007/s11548-020-02288-8 (PMC7880982; doi:10.1007/s11548-020-02288-8)
Supplement: Supplementary file 1 — Supplementary material 1 (DOCX 49 kb) [file 11548_2020_2288_MOESM1_ESM.docx]

**Supplement Table 1**

| Supplement Table 1. Standardized segmentation of anatomical structures: methods and grey value thresholds [3]. | | | |
| --- | --- | --- | --- |
| **Structure** | **Method** | **Minimal threshold** | **Maximal threshold** |
| Skull | Thresholding | Undefined | 788 |
| Stapedius muscle | Threshold-masked painting | Undefined | 850 |
| Facial nerve | Threshold-masked painting | 0 | 850 |
| Inner ear | Threshold-masked painting | 1 | 1000 |
| Sigmoid sinus | Painting & morphological contour interpolation | Undefined | Undefined |

**Supplement Table 2**

| Supplement Table 1. Comparison between the Experts’ opinion from Volk et al. [[3](#_ENREF_3)] and the measurements of the surgical planning tool. | | | | | | | | |
| --- | --- | --- | --- | --- | --- | --- | --- | --- |
| **Experts’ opinion** | **SM exposed area in % of total SM area** | | **Diameter of the surgical corridor at the plane of the mastoid in mm** | | **Distance SM-FN in mm** | | **Depth of SM behind FN in mm** | |
|  | Mean | SD | Mean | SD | Mean | SD | Mean | SD |
| SM only partially exposed | 43.20 | 28.18 | 8.31 | 6.11 | 0.89 | 0.18 | 1.97 | 1.48 |
| SM exposed | 63.81 | 25.44 | 12.00 | 8.24 | 1.03 | 0.44 | 1.52 | 1.04 |
| Access not feasible | 15.20 | 21.98 | 6.60 | 4.24 | 1.01 | 0.35 | 1.59 | 0.06 |
| Access feasible | 57.00 | 27.88 | 10.92 | 7.62 | 0.99 | 0.37 | 1.69 | 1.24 |

SD = standard deviation; SM = stapedius muscle, FN = facial nerve
